# Implementing A person-centred CommunicaTION (ACTION) educational intervention for in-home nursing assistants – a study protocol

**DOI:** 10.1186/s12877-023-03831-3

**Published:** 2023-02-25

**Authors:** Jessica Höglander, Inger K. Holmström, Tanja Gustafsson, Elisabeth Lindberg, Hanna Maurin Söderholm, Lena Hedén, Petra von Heideken Wågert, Annelie J. Sundler

**Affiliations:** 1 School of Health, Care and Social Welfare, Mälardalen University, Västerås, Sweden; 2 Department of Public Health and Caring Sciences, Uppsala University, Uppsala, Sweden; 3 Faculty of Caring Science, Work Life and Social Welfare, University of Borås, Borås, Sweden

**Keywords:** Competence development, Education intervention, Home care, Nursing assistants, Older persons, Person-centred communication, Study protocol

## Abstract

**Background:**

In this study, the focus is on how to support the competence development needed for nursing assistants in home care. Home care services for older persons can be challenging concerning the nature of the interpersonal interaction and communication needed to care for and respond to the diverse needs of older people who seek to live well in our communities. This implies a need to offer more person-centred care (PCC) to older persons. However, there is a lack of knowledge on how to develop such competence. We, therefore, developed A Person-centred CommunicaTION (ACTION) programme, which is a web-based educational intervention aimed at supporting competence development for nursing assistants. The research objective is to evaluate the ACTION programme with respect to participants’ responses to and the effect of the intervention.

**Methods:**

A multicentre case–control study with pre- and post-assessments was designed. The ACTION programme will be implemented at home care units, in two different geographic areas in Sweden. A total of 300 nursing assistants will be recruited: 150 for the intervention group and 150 for the control group. We will evaluate the impact measures and the process. Pre- and post-assessments will be performed with data collected via a) audio recordings of communication, b) a questionnaire on self-efficacy communication skills, PCC, empathy and job satisfaction, c) user data, evaluation forms, field notes and observations, and d) interviews. The data will be analysed with descriptive and analytic statistics and/or qualitative methods for meanings.

**Discussion:**

This study has the potential to contribute to the evidence supporting competence development required to offer person-centred and quality home care to older persons and to meet upcoming needs for flexible and easily accessible competence development.

**Trial registration:**

ISRCTN64890826. Registered 10 January 2022, https://www.isrctn.com/ISRCTN64890826

## Background

This study protocol presents the implementation of A Person-centred CommunicaTION (ACTION) programme targeting nursing assistants (NAs). NAs must have the competence needed to care for and respond to the diverse needs of older people who seek to live well in their communities. Home care services are a part of Sweden’s welfare system, delivering health and care services at home, and supporting older persons to remain living in their homes for as long as possible. Home care services are foremost delivered by NAs, who are one of the largest occupational groups in Sweden [1]. They have a prominent position, performing most patient/client-direct care to older persons at home. The workload and work complexity have increased for NAs, and the Swedish welfare system is facing challenges in keeping competent staff for the care of older persons and in supporting good working conditions [1, 2]. The recruitment and retention of competent staff with the skills needed for quality care for older persons is a future challenge. Therefore, we developed the ACTION programme to support competence development in home care for older persons.

### Current challenges related to individualised care and the significance of person-centred communication skills

The home care of older persons is multifaceted and can be challenging concerning the nature of interpersonal interaction and communication needed to succeed with care actions and to support older persons’ dignity and well-being. Home care is important for supporting healthy ageing for older persons living at home and needs to build on communication and interaction that shows respect and dignity while acknowledging individual needs. The research suggests that communication and person-centred care (PCC) are particularly helpful to uphold dignity, empathy, well-being, and humanity in the care of older persons [3–5]. The communication and interaction between older persons and NAs are important for a successful outcome of individualised care [6]. A positive relationship with time for individual concerns is supportive and can demonstrate trust and empathy. These factors also affect an individual’s self-management of illness [7] and thereby health and well-being.

Considering the interpersonal nature of individualised care, the communication skills of staff become a key competence. While communication occurs naturally during nursing activities, research suggests that everyday conversations can be used to elicit information, normalise unpleasant procedures and manage interactions and relationships [8]. However, communication and interaction are subjected to challenges due to older persons’ fragility, existential concerns, and emotional needs, which are commonly vaguely expressed and difficult to notice [4, 8, 9]. To successfully manage various caring situations, the competence of individual NAs is crucial. Thus, skills in person-centred communication are vital for the individualised care of older persons with diverse needs and circumstances. Feasible interventions with better utilisation of competence are required.

### Work complexity and challenges related to key competences of NAs

The work complexity of home care of older persons can result in challenging situations for NAs related to communication and ethical or existential concerns [4, 8–10]. There might also be difficulties in organisational structures [4, 10], for instance, strict rules, time limits and regulations of home care visits. Moreover, changes in the welfare system related to rationalisation and marketing make NAs’ work more standardised and detailed, leading to less ability to control their daily work [11]. In contrast, research indicates that PCC can have a positive impact on job satisfaction [12]. In addition, improving communication skills among staff can also benefit both patient and staff outcomes [3].

There are approximately 135 000 NAs in municipality home care or nursing homes in Sweden [1]. Most of them have vocational training from high school health education and a majority are women (91%). The number of employees without training or formal college or university education is estimated to be lower than those with training. A relatively large proportion of employees in municipal care are reported to be low qualified, and some staff lacks sufficient competency to ensure that older persons receive the care they require [13, 14]. In the near future, before 2028, over 35 000 NAs will reach retirement age [1]. Hence, there are challenges in ensuring the right competence of staff in the municipal care of older persons.

Competence is a complex concept defined as a combination of skills, knowledge, attitudes and abilities [15]. Competence development in this area requires a pedagogical approach integrating education with personal experiences and abilities. Educational support is needed for NAs to meet the individual needs of older persons [16].

### Previous implementation of person-centred care or communication interventions

Currently, PCC is implemented in a variety of healthcare contexts [5]. There is an ongoing study with a focus on the effect of education on PCC for nursing staff in nursing homes [17]. However, there is no previous study with a focus on person-centred *communication* tailored to home care, even though there have been some communication intervention studies in other healthcare settings [3, 18–21]. Previous studies on implementing person-centred and empathic communication in home care are scarce. In addition, interventions in home care are complex, not least related to the work complexity and challenges in sustaining competent staff needed for individualised quality care of older persons. More research is necessary on how to successfully implement and evaluate educational interventions with suitable approaches and components for NAs in home care. Therefore, we developed the ACTION programme for competence development targeting NAs in home care. The research objective is to evaluate the full-scale implementation of the ACTION programme with respect to participants’ responses to and the effect of the intervention.

### Theoretical frameworks of the ACTION programme

#### Development of the educational intervention

The ACTION programme was developed to address the challenges of keeping and safeguarding competencies imperative for quality home care services and to meet current and upcoming needs for flexible and easily accessible education. A participatory-driven design for co-creation, involving NAs, managers, and researchers, was used. The programme combines the essential competences of PCC and communication for the development of NAs’ expertise, needed for high-quality person-centred care and the well-being of older persons. ACTION is a short, mainly web-based education with a clear focus on the practical implementation of person-centred communication, influenced by previous research in this field and from the researchers’ previous studies in the COMHOME project [22]. Different pedagogical approaches are used to support the practical use of this knowledge when caring for older persons in their homes. The intervention is designed to be flexible, accessible, and easy to implement. The programme was developed based on four core components: I) knowledge of key competence in communication and person-centred care, II) self-directed and reflective learning, III) flexibility and accessibility, and IV) efforts to stimulate and encourage participant activity [23].

The ACTION programme was first evaluated in a small-scale feasibility study [23]. The evaluation of the small-scale intervention showed promising results. The findings emphasise the benefits of a web-based learning format and the critical importance of organisational support and available resources. A relatively high level of participation by NAs was observed. This might indicate that the education was found to be meaningful. The web-based format was easy to use, and the NAs appreciated the flexibility and accessibility. After this small-scale feasibility study, some refining work has been made based on the findings.

#### Implementation strategy

The implementation strategy is tailored to attributes known to be important and to allow the intervention to fit local circumstances. Facilitation is considered important, and we involved NAs and managers to obtain suggestions and feedback. To achieve the best possible outcomes, with respect to participation and acceptance, the implementation work is guided by Everett Rogers [24] in combination with the work by Boaz et al. [25]. The guiding principles and strategic attributes for successful implementation are to provide a relative advantage compared to other strategies (e.g., traditional classroom education); compatibility with home care practice and work organisation; and to minimise the complexity (i.e., easy to understand and access). This is combined with a flexible and web-based approach [25] by using a web-based learning platform designed for education. To ensure accessibility, the research team will provide support and different solutions to allow participants access either through computers at their workplace, tablets provided by researchers, or the participants' mobile phones. Facilitators will support their participation, together with weekly e-mail reminders and newsletters. We are aware of differences in technical skills among the participants. The learning platform is widely used by students in higher education in Sweden, is considered easy to use, and requires a limited amount of IT experience. The researchers will ease any technical problems and support the NA’s access. Close collaboration between researchers and individuals within the home care units will be prioritised and is considered imperative for implementation.

#### Theoretical framework for person-centred communication

Our approach to person-centred communication builds on a combination of theories on PCC and communication. The theoretical point of departure for PCC is the theory and philosophy of psychologist Carl Rogers [26]. Carl Rogers emphasises a caring approach based on principles and values of acceptance, caring, empathy, and sensitivity in human interactions [26]. PCC and communication are intricately linked and widely recognised as important concepts in quality of care. The literature on PCC describes communication, respect, autonomy, empathy, and empowerment as fundamental components [27–29]. PCC reflects humanistic values of respect for the person and individual rights to self-determination, with mutual respect and understanding [30]. The ability to be sensitive and listen is essential, which includes the acknowledgement and confirmation of emotions [31]. The communication of care providers is an important competence to facilitate PCC, and PCC can be accomplished and supported by communication competencies. There are, however, challenges in achieving PCC in reality.

In addition to the framework of PCC, the basis for communication is built on the theory by Watzlawick et al. [32] and partly on the model for empathic communication by Suchman et al. [33]. Communication and interaction between people have two main functions: exchanging information and building relationships. Communication is a complex process and is defined as the act of transmitting meanings and messages between people. Everything one does is a message: actions, words and silences all have a message [32]. Emphatic communication can be understood as sequences of interactions with emphatic opportunities. A basic skill for being empathic is to recognise patients’ expressions of emotional concerns, even if not explicitly expressed, to invite further exploration while acknowledging the emotions and making the patients feel confirmed and understood [33]. Recognising emphatic opportunities can help improve empathic responses with an impact on care quality and satisfaction for patients and staff.

#### Pedagogical strategies

**Fig. 1:**
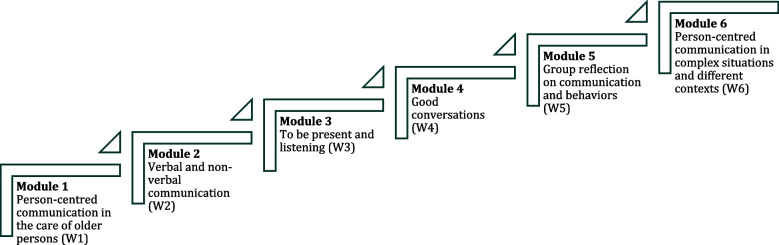
Overview of the stepwise education modules during the six educational weeks (W1-W6)

The ACTION programme has a blended learning approach, combining e-learning and face-to-face instructions [34]. Our pedagogical strategies aim to support a self-directed and reflective learning approach. By using flexible and experience-based learning, the goal is to stimulate and encourage NAs to learn and mature in their person-centred communication skills. This is in line with Knowles's [35] theory of adult learning, highlighting the importance of a self-directed learning approach with reflection. To develop knowledge and mature in one’s understanding, previous work experiences can be a resource for learning. The pedagogical strategies are adopted in the ACTION programme, and the education is structured into six stepwise modules, combining important components of PCC and communication, please see Fig. 1. These modules contain a combination of web-based lectures, quizzes, reflection assignments and one on-site group supervision. The theoretical content includes knowledge on, for example, careful listening, asking questions, being attentive and empathic, and verbal and nonverbal communication.

## Methods

### Design

The ACTION programme is designed as a multicentre case–control study with a pre-post, intervention design. Previous research has stressed a need for further controlled studies to examine the tendency observed in individual studies [3]. Implementing interventions, such as the ACTION programme in home care, is complex as several components can interact and affect each other. Therefore, our research is guided by the framework for complex interventions [36], combined with a multifaceted implementation strategy [25]. Both implementation outcomes and intervention outcomes will be evaluated [36], and the STROBE guidelines for the conduct and dissemination of observational studies will be used [37].

**Fig. 2:**
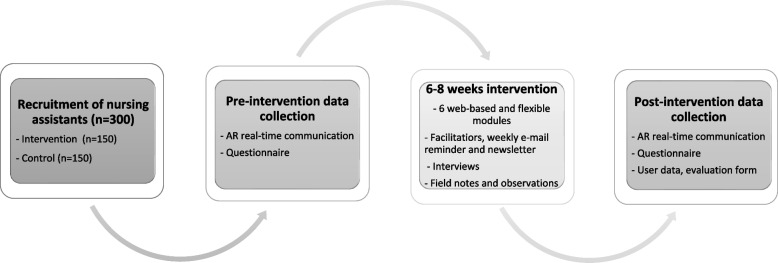
Overview of the intervention and data collection

Qualitative and quantitative measures will be combined, and multiple data from audio-recorded real-time communication, questionnaires and interviews will be collected and analysed. The settings and samples for the project are home care units located in western and mid-Sweden, in both urban and rural areas. The intervention will be conducted during the regular work of the NAs and will not affect the regular care. For an overview of the intervention and data collection, please see Fig. 2.

The ACTION programme is a collaboration between the University of Borås and Mälardalen University, but is initiated by the University of Borås, who also is primarily responsible for the study.

### Settings and samples

Approximately 300 NAs will be recruited for the study. Participants will be randomly assigned to either control (*n* = 150) or intervention group (*n* = 150). The recruitment will be organised into two stages: the first stage will be performed by researchers at University 1 with the recruitment of 75 participants in each group, e.g., intervention versus control group. In the next stage, researchers at University 2 will repeat the procedure with the recruitment of 75 participants in each of the intervention and control groups. The power calculation estimated that a sample size of 115 NAs was needed in each group to capture a statistically significant difference in outcomes after the intervention, calculated with a rate ratio of 1.5 with 80% power and a Type I error rate of 5%. The estimation of the sample size is based on the hypothesis that NAs in the intervention group will have a greater number of empathic responses after intervention than those in the control group. To account for dropouts, this number was extended to 150 NAs in each group.

Home care units will be recruited based on contacts with managers who are willing to implement the educational programme for NAs at their unit. A researcher from the project will visit the home care units and provide information about the ACTION programme to the NAs. Every NA, at each home care unit, will be offered to participate in the ACTION programme. After informed consent, they will be included. All participating home care units will be blinded, and do not know if their unit will be part of a control or an intervention group. The randomisation is done by recruiting every second unit to the intervention versus the control group, to aim for an even distribution of the number of units in each group. The intervention groups will be offered the education during implementation, and the control groups will be offered the education after completion of all data collection to compensate for their effort to collect data as controls.

### Data collection

Pre- and post-assessments will be performed with data collected on:

- a) Audio recordings (ARs) of real-time communication between older persons and NAs during home care visits before and after the intervention, e.g., 1 plus 1 per NA;
- b) a questionnaire on self-efficacy communication skills, PCC, empathy, and job satisfaction;
- c) user data, evaluation form, field notes, observations, and;
- d) interviews.

To evaluate the effect of the intervention on NAs’ communication, data will be collected by ARs and questionnaires. ARs are considered important data sources when conducting in-depth analyses of interpersonal communication [38]. ARs of real-time communication allow for the collection of empirical data on authentic communication during home care visits, which makes it possible to capture communication in its natural context. Such observations are suggested as an efficient and highly valued method for data collection that can facilitate powerful data and provide new insights into communication in nursing practice, different from interviews where gaps may occur between what people report and recall about communication and behaviours during nursing care [39]. Hence, a strength in ACTION is the evaluation of the actual communication by ARs. There are, however, challenges when recording actual situations. For example, participants may be aware of being recorded and change their behaviour [40], but no major differences have previously been observed [41, 42]. It is the NAs who handle the ARs themselves, no researchers will be present during the home care visits. In order to do this, the NAs will receive thorough information on how to handle the ARs.

All NAs are also expected to complete questionnaires on self-efficacy of communication skills, PCC, empathy, and job satisfaction (see Table 1). Participants in the control group will be asked to complete the same measures at approximately the same time.

**Table 1:** Questionnaires used in the ACTION programme

Questionnaire	Items and scale	Focus
Self-efficacy questionnaire (SE-12) [43]	12 items 10-point Likert scale	Confidence in clinical communication skills of healthcare professionals
Person-centred Care Assessment Tool (P-CAT) [44]	13 items 5-point Likert scale	Nursing staff rating the extent of person-centred care in care settings
Jefferson Scale of Empathy (JSE) (Health-provider version) [45]	20 items 7-point Likert Scale	Empathy among health professionals in clinical practice
Measure of Job Satisfaction [46]	38 items 5-point Likert scale	Job satisfaction among nurses: Personal Satisfaction; Satisfaction with Workload; Satisfaction with Professional Support; Satisfaction with Pay and Prospects; and Satisfaction with Training

Data collected with interviews, field notes, and observations will contribute qualitative aspects and experiences during implementation and are important for identifying and resolving potential problems. To evaluate the benefits and potential barriers to implementing competence development in person-centred communication with NAs in home care, we will collect user data on NAs’ participation from the web-based platform, anonymous evaluation forms, field notes and observations. An example of user data is information about when and for how long the NA has visited the learning platform. This will be done by monitoring information logs available through the learning platform and written evaluations from the NAs. Only the researchers within the ACTION programme will have access to the information logs. No information regarding the NAs' participation in the learning platform will be shared with their employers or any other unauthorised person. Field notes and observation notes will be written by the researchers for documenting the implementation process, for example, any strengths or weaknesses in the design or content of the learning modules, the ease of use of the digital learning platform, unforeseen challenges that may arise and important lessons learned for future educational interventions. No personal information about the NAs will be kept in the field or observation notes.

In addition, older persons’ perceptions about NAs’ person-centred communication skills will be collected through individual semi-structured interviews with 20 older persons receiving home care during the implementation phase. It is difficult to estimate how many participants will be needed, thus, the estimation of 20 older persons may be reduced if saturation is reached [47]. The individual interviews with older persons will contribute information about their experiences of communication with NAs during home care visits.

### Outcome measures

Demographic data on NAs and older persons will be collected. NAs will be asked to self-report characteristics regarding sex, age, native language, and work experience. Older persons recruited for ARs and/or interviews will also be asked to report on sex, age, and native language.

The following measures will be used to evaluate the effect of the intervention on NAs’ communication competence and for process evaluations:

#### Primary outcome measure

- a) *Person-centred communication* will be measured with audio recordings of communication during home care visits collected at baseline (e.g., before education) and after education (e.g., 8 weeks later) and analysed and coded by sequences of empathic statements and responses to emphatic opportunities, emotional communication, and degree of person-centredness.
  - i. Emphatic and emotional communication, defined according to empathic opportunities, is described by Suchman et al. [33] and their model of emphatic communication.
  - ii. Emotional communication is coded by the Verona Coding Definitions on Emotional Sequences (VR-CoDES) [48].
  - iii. Degree of person-centred communication coded by the Roter Interaction of Analysis (RIAS) [49].

The coding will be made by two independent coders after training, and interrater reliability will be calculated with Cohen’s kappa coefficient or with Pearson correlation analysis.

#### Secondary outcome measures

- b) *Communication skills* [43]*, Person centred care* [44]*, Empathy* [45], and *Job satisfaction* [46] will be measured with questionnaires at baseline (e.g., before education) and after education (e.g., 8 weeks later).
- c) *Process evaluation* will be conducted with interviews, evaluation forms, field notes, and observations during the education with a focus on qualitative aspects and experiences of the implementation, weekly at weeks 1–6 during the education.
- d) Older persons’ *perceptions about the meaning of and experiences with* NA’s person-centred communication will be measured with interviews, at weeks 6–8.

### Coding systems and questionnaires

#### Empathic opportunities

The coding system defines terms for describing patients’ expressions of emotions and the provided response from the healthcare provider. There are seven defined terms: empathic opportunities, empathic response, empathic opportunity terminator, missed empathic opportunity, potential empathic opportunity continuer, and potential empathic opportunity terminator [33, p.679]. In addition, opportunities for praise can be defined in terms of praise opportunity, praise, and praise opportunity terminator [33, p.681].

#### VR-CoDES

VR-CoDES can be used for coding opportunities of emotional communication in ARs by coding patients’ utterances of emotional distress and the healthcare providers' responses to these utterances. The coding system contains detailed codes for both utterances of emotional distress and subsequent responses to these utterances. Emotional distress is coded either as a clear expression of an emotional concern or as an implicitly expressed cue of a patient’s emotional distress, and there are seven definitions of cues [48]. After a concern/cue, the healthcare providers’ response is coded, either as an explicit or nonexplicit response, which provides or reduces space for further disclosure and is then divided into further definitions of response categories (a total of 17 categories) [50].

#### RIAS

Originally, RIAS was constructed to code medical dialogue, but it has been widely used in other areas of communication research [49]. RIAS focuses on establishing task-focused and socioemotional elements in communication. These elements are established by assigning codes to the communication. From the coded elements, a ratio of patient-centeredness, in the communication, can then be calculated. RIAS has a total of 40 codes, which are applied to the smallest unit of expression to which a code can be assigned, for example, a sentence, a section of a sentence, or a single word.

#### Questionnaires

In addition to demographic data about the participants, surveys obtained through questionnaires will contribute self-reported information regarding communication skills and abilities, empathy, and job satisfaction. All questionnaires have been validated and used in other studies and are published elsewhere. Please see Table 1 for information regarding the questionnaires in the ACTION programme.

### Analyses and data processing

Data will be analysed with quantitative and/or qualitative methods. Descriptive statistics will be used for participants’ characteristics to present findings of the coding of empathic opportunities, reports on self-efficacy communication skills and job satisfaction, and data on participants' adherence.

For analysis of our primary outcome, we will compare the number of empathic statements and the number of responses to an empathic opportunity with a continuer rather than a terminator, similar to the study by Tulsky et al. [51]. The number will be calculated in pre- and post-assessments and compared between the control and intervention groups. Considerations will be made regarding hierarchical and clustered data, e.g., both the NAs and older persons may be recorded several times. For the analysis of differences between the intervention and control groups, we will consider using a generalised linear model form of regression analysis. For statistical analysis of differences between subgroups, chi-square tests will be used for responses given on nominal scales and independent t-tests or corresponding nonparametric tests if needed for responses given on continuous scales.

For analysis of secondary outcomes, all self-reported measures from the survey will be treated as continuous variables and compared between the intervention and control groups. Descriptive statistics will be used to analyse the answers reported. We will consider subgroup analysis with inferential statistics on differences related to sex, age, and native language. The analysis will follow the same statistical analysis as described above for the primary outcome. All statistical analyses will be two-sided and performed in IBM SPSS Statistics software Version 25.

Data on field notes and reports from participants will be collected as text, and each interview will be transcribed before analysis. Both field notes, interviews, and reports will be analysed qualitatively with content analysis [52]. The content analysis will be inductive and grounded in the actual data, i.e., conventional content analysis. Conventional content analysis is appropriate when previous research or theories are limited. The data will be read several times to gain a sense of the whole. The content will then be organised into codes and sorted into categories according to differences and similarities that emerge from the data. Exemplars for each category will be presented when reporting the results. The advantage of using conventional content analysis is that the information is gained directly from the data without interpretation, theoretical perspectives, or predetermined categorisations [52].

### Dissemination

The ACTION programme and its findings will be disseminated and communicated through universities’ web pages and social media about progress and findings. The translation of research findings into clinical practice continues to be one of the most challenging phases of the research process. We will actively promote the dissemination of findings, using lay language to the general community and users, as well as care providers, managers, and clinical networks at a local and national level. This will involve presentations and seminars within organisations and with professional networks and arenas. In addition, the project and findings from our research will be spread in national and international networks, conferences, and meetings, and distributed through web pages and social media. The dissemination of findings to the scientific community will mainly be addressed to an international audience through scientific publications in peer-reviewed journals.

## Discussion

**Fig. 3:**
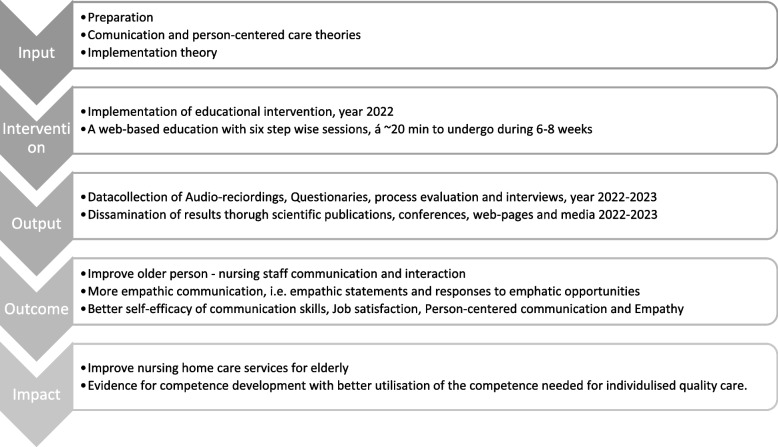
Overview of the development and outcome of the ACTION programme

The proposed study builds on a small-scale feasibility study [23] with promising results on the benefits of the web-based learning format being easy to use and the NAs appreciating the flexibility and accessibility. The present study has the potential to contribute to the evidence supporting key competence development required to offer individualised care for older persons and to meet upcoming needs for flexible and easily accessible competence development. There are few risks connected to the study, however, both ARs of home care visits and interviews can be perceived as uncomfortable for the individuals’ privacy. For example, ARs can be sensitive and uncomfortable for both NAs and older persons who receive home care. Possible benefits are designing flexible and easily accessible web-based education within a complex and multifaceted care context and developing competence in person-centred care for NAs caring for frail older people in the community. For an overview of the development, proposed outcomes, and impact of the ACTION programme, please see Fig. 3.

Feasible interventions are required to enhance PCC and communication competency needed for continuously improved home care services. However, challenges have been identified, and considerations are needed when conducting a complex intervention in the actual home care setting. The development of the ACTION programme has therefore been preceded by a feasibility study [23], assessing the intervention before any large-scale implementation. Assessing the intervention is sensible and important since there is no guarantee that the intervention will be accepted or sufficiently delivered to the intended recipients [53].

One identified challenge is related to the researchers’ access to the field, involving participants and the adherence of participants to the intervention. Hence, local facilitators should be assigned early when planning for the implementation and will function as support and contacts during the implementation, mediating the communication between the researchers and the NAs [23]. Other challenges are related to measures of study outcome (i.e., the communication competence of NA) since no feasible instruments have been found in previous studies that are suitable to measure the outcome of the communication intervention. However, a strength of this study is the evaluation of the actual verbal communication of home care visits by ARs and through coding empathic statements and opportunities, exploring the person-centred content of the communication. Nevertheless, the analysis of the ARs is restricted to empathic opportunities and the presence of person-centred aspects in the communication, whereas other important outcome measures for evaluating the effects of the intervention may be missed. However, considerations are made according to previous interventions in similar settings, and the researchers are motivated to use methods like those used in the study by Tulsky et al. [51] and found in the systematic review by Kerr et al. [54]. Hitherto, interventions with a focus on communication are sparse, which may be due to difficulties in assessing outcome measures. Hence, further studies and methods need to be developed in this area.

One main concern in ACTION is to make education feasible, that is, accessible and applicable. Blended learning is chosen as a pedagogical strategy within ACTION, combining e-learning and face-to-face interaction, and can be seen both as flexible and accessible. Blended learning may also have its challenges, such as how much flexibility is desirable, how to facilitate interactions between learners and instructors, how to facilitate a learning process when based on self-regulation, and how to create a good learning climate [55]. Learning in ACTION is self-regulated, which means that the participants themselves are responsible for their learning process. Therefore, using clear instructions, time frames, weekly newsletters, and reminders, become important to facilitate the participants' learning process. Instructors and peers will function as facilitators (external and internal) to support the participants’ learning, and instructors can monitor participants’ digital activities to follow up on those who are inactive. The introductory face-to-face meeting in the first module and a face-to-face group session, later on, can provide opportunities for interaction with instructors and participants (i.e., learners). This is in line with the evaluation results from the feasibility study by Gustafsson et al. [23], where the participants valued face-to-face interactions and reflections together with peers. Positive outcomes of using an introductory face-to-face meeting are also outlined in a previous literature review [55], hence reducing some of the challenges regarding learning processes, interaction, and climate. However, some evidence suggests that web-based education on communication is sufficient and can improve healthcare professionals’ communication self-efficacy [19]. Nevertheless, NAs need to find the intervention useful and relevant and experience satisfaction with its content and design; otherwise, there might be a risk of having a negative effect on the outcomes of the implementation.

## Data Availability

Not applicable.

## Abbreviations

AR: Audio recording
NA: Nursing assistant
PCC: Person-centred care
